# Post-traumatic stress disorder and associated factors among people who experienced traumatic events in Dessie town, Ethiopia, 2022: A community based study

**DOI:** 10.3389/fpsyt.2022.1026878

**Published:** 2022-10-25

**Authors:** Tamrat Anbesaw, Yosef Zenebe, Amare Asmamaw, Maregu Shegaw, Nahom Birru

**Affiliations:** Department of Psychiatry, College of Medicine and Health Science, Wollo University, Dessie, Ethiopia

**Keywords:** Dessie town, post-traumatic stress disorder, depression, Ethiopia, stress

## Abstract

**Background:**

Posttraumatic stress disorder (PTSD) may develop as a serious long-term consequence of traumatic experiences, even many years after trauma exposure. Dessie town residents have experienced prolonged armed conflict due to inter-communal conflict in 2021. Those people are exposed to different kinds of trauma, and violence, making them more prone to psychological disorders. Despite the highest number of people affected due to conflict and its negative impact on mental health, post-traumatic stress disorders among people are overlooked in Ethiopia. This study aimed to assess the prevalence and associated factors of post-traumatic stress disorder among people who experienced traumatic events in Dessie town, Ethiopia, 2022.

**Materials and methods:**

Community based cross-sectional study was conducted on June 8–July 7, 2022, by using a multi-stage cluster sampling with a total sample of 785. Pretested, structured questionnaires and face-to-face interviews were used for data collection. PTSD was assessed by the Post-traumatic stress disorder Checklist for DSM-5 (PCL-5). Data was entered using Epi-data version 3.1 and, then exported to SPSS version 26 for analysis. The association between outcome and independent variables was analyzed with bi-variable and multivariable logistic regression. *P*-values less than 0.05 was considered statistically significant.

**Result:**

The prevalence of PTSD among Dessie town residents was found to be 19.4% (95% CI, 16.7, 22.0). In multivariable analysis, being females (AOR = 1.63, 95% CI 1.10–2.44), previous history of mental illness (AOR = 3.14, 95% CI 1.14–7.06) depressive symptoms (AOR = 3.12, 95% CI 1.92–5.07), witnessing a serious physical injury of a family member or friend (AOR = 2.82, 95% CI 1.18–6.70) and high perceived life threats (AOR = 5.73, 95% CI 3.05–10.78) were found to be significant predictors of PTSD.

**Conclusion:**

The prevalence of PTSD among Dessie town residents was found to be huge. Being female, having a previous history of mental illness, depressive symptoms, witnessing a serious physical injury of a family member or friend, and high perceived life threats were variables that are independent predictors of PSTD. People who have experienced such a severe traumatic event require psychosocial support to aid in their recovery from the terrible experiences.

## Background

According to the fifth edition of the Diagnostic and Statistical Manual of Mental Disorders (DSM-5), Posttraumatic stress disorder (PTSD) is one of the mental disorder related to the following exposure to a traumatic or stressful event which is characterized by the symptoms of intrusion, avoidance, alternation of mood and cognition and hyperarousal lasts for more than a month after the stressful event (1). Experiencing or witnessing traumatic events including murder, threats, kidnapping, the death of loved ones or friends, the loss of a home, or malnutrition can lead to PTSD (2). The global economic burden of stress-related mental illness is expected to rise in the coming decade (3). The world health organization (WHO) global disease of burden survey estimates that mental illness, including stress-related disorders, will be the second leading cause of disability by the year 2023 (4). In a given year, PTSD affected about 8 million adults worldwide (5). PTSD was followed by 0.4% of all years lived with disability, and the estimated burden increased to 0.6% of years of healthy life lost due to disability (YLD). globally, according to the world health report (3). In the upcoming decade, it is anticipated that trauma-related mental illness will have a greater economic impact and raises the risk of physical illness (diabetes, obesity, pain, etc.) globally, leading to disability and compromising the quality of life.

In post-conflict and conflict-ridden societies, the prevalence rates in the general population can be much higher (6). According to statistical estimates, the prevalence of PTSD ranges from 1 and 5% in studies of the general population (7, 8), and from 3 and 58% in high-risk groups such as conflict areas (9, 10). Northern Ethiopia went through one of the worst civil conflicts that attracted the world’s attention in 2021. The civil conflict was between the Ethiopian National Defence Forces (ENDF) and The Tigray People’s Liberation Front (TPLF) party (11). Due to this thousands of people died, and many people were injured and were subjected to traumatic events due to this conflict while they were trying to survive. Additionally, nearly 2 million people were displaced into internally displaced people’s camps (IDPs) as a result of violent threats that included rape, torture, mutilation, and destruction of property and many of them people including children were abducted, rendering them more susceptible to psychological disorders, particularly post-traumatic stress disorder (PTSD) (11).

A meta-analysis study conducted on a global population of adult war survivors that looked at all countries that suffered at least one war within their territory between 1989 and 2015 found that 23.81% of adult war survivors met the diagnostic criteria for PTSD (12). Another systematic review undertaken revealed that the prevalence of PTSD in the community ranges from 3 to 88% (13, 14). In addition, a cross-sectional survey was conducted among community levels in Nepal during armed conflict and the reported prevalence of PTSD was 53.4% (15). In another research conducted on Palestinian people who were living in refugee camps during the Al-Aqsa intifada, the estimated prevalence of post-traumatic stress disorder was 68.9% (16). Furthermore, studies showed in Africa, internally displaced victims in Nigeria 63% (17), Morocco 19.3% (18), and South Sudan 28% (19). Some studies report from Ethiopia showed Maikadra Massacre Suffer in North West, Ethiopia 59.8% (16), and internally displaced people in South Ethiopia 58.4% (20). Another study finding from West of Ethiopia showed the overall prevalence rate of PTSD among traumatic Patients was 17.1% (21). In a community-based, cross-sectional study conducted on landslide survivors, in Addis Ababa, Ethiopia the prevalence of PTSD was 37.3% (22).

Many studies have shown a link between PTSD and various risk factors before the injury like sex, low educational status, low social support, unemployment, lower socioeconomic status, younger age, childhood abuse, history of depressive symptoms, and history of mental illnesses (23–25). Also, factors during the traumatic events such as witnessing the death and serious physical injury of a family member or friend, the property being destroyed during the conflict, serious physical injury during the conflict, and alcohol use (12, 16–18, 20, 22, 26). Additionally, factors after the injury like high perceived life threats, and the property being destroyed after the conflict (16) have been frequently reported as predictors of posttraumatic stress disorder.

Conflicts reportedly killed over three times more people during the past decade than natural disasters (27). In Ethiopia, where armed conflict, ethnic violence, and terrorist attacks are on the rise, only a few studies with highly variable estimates of the prevalence of post-traumatic stress disorder have been published, despite mounting evidence of its high prevalence in conflict-affected nations around the world. Moreover, it was reported that the number of reports that dealt with the prevalence of mental illnesses among people who were living in areas of conflict is limited. Therefore this study is aimed at assessing the prevalence of post-traumatic stress disorder and its predictors among people who experienced traumatic events in Dessie town situated in the area of armed conflict. The findings of this study will help health professionals, NGOs, and psychological centers to develop appropriate plans and interventions to provide evidence-based treatment for patients with PTSD. Additionally, it can also serve as baseline data for those who wish to conduct studies in this area.

## Materials and methods

### Study setting, design, and period

A community-based cross-sectional study was conducted in Dessie Town, Northeast Ethiopia, from June 8 to July 7/2022. The administrative center of Dessie Town in the Amhara Region; is located from Addis Ababa to Northeast Ethiopia 401 Km. It sits at a latitude and longitude of 11°8′N 39°38′E, with an elevation between 2,470 and 2,550 meters above sea level. It has 18 kebeles and has a 350,000 population. Among those populations 186,571 males and 163,429 females according to 2016 to 2017 South Wollo Zone statistics office data. The two governmental hospitals have psychiatric outpatient and inpatient services.

#### Source of population

All residents of Dessie town, North East, Ethiopia.

#### Study population

Households live in the Menafesha sub-city who are in the selected kebele and available during the study period.

### Inclusion and exclusion criteria

All households in selected kebeles and one individual from each household who was 18 years old and above, living in Dessie town during the time of the study were included, while participants who were not available during wartime, severely ill, and unable to communicate during data collection time and those residents less than 6 months were excluded from the study.

### Sample size determination and sampling technique

#### Sample size determination

To calculate the maximum estimated sample size, the single population proportion formula was used at 95% CI and 5% marginal error and by taking the *P*-value of 58.4% from the previous study in Southern Ethiopia (20). Then the sample size was calculated as follows;


$$\text{n}=\frac{{\left(Z\,\alpha /2\right)}^{2}\,P\,\left(1-P\right)}{d^{2}}$$

where: n = sample size, P = prevalence of previous study (58.4%), d = degree of precision (assumed to be 5%), (z α/2)^2^ = 1.96 = the value of standard normal variable that corresponds to be 95% confidence levels (1.96).


$$\text{n}=\frac{{\left(1.96\right)}^{2}\times [0.584\left(1-0.584\right)}{{\left(0.05\right)}^{2}}=373$$

Since we have employed a multi-stage sampling technique to consider the designing effect, the calculated sample size by two to correct the sampling error. After all, by adding a 10% non-response rate, the final sample size was 821.

#### Sampling technique and procedure

**FIGURE 1:**
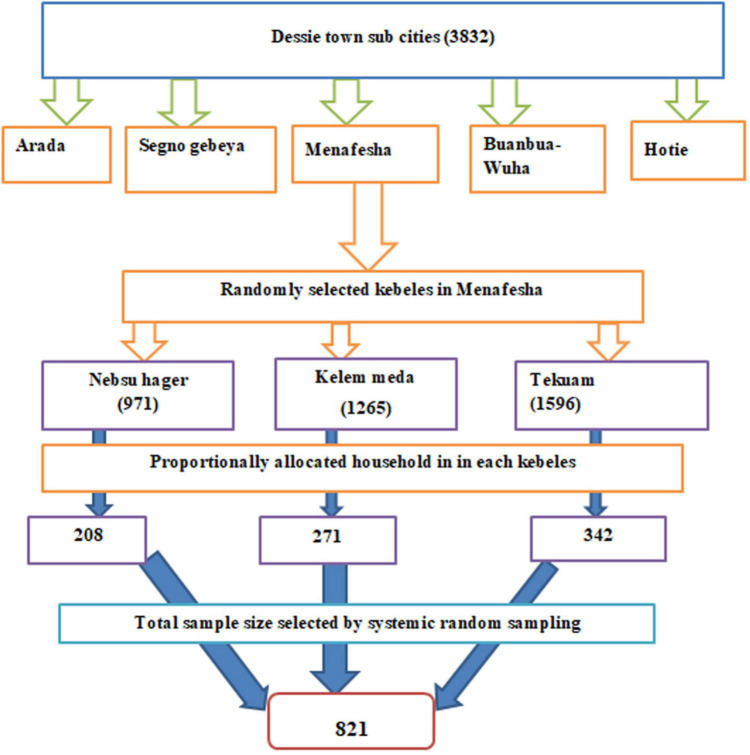
Schematic representation of sampling technique showing the number of selected samples from each sub-city of Dessie city, 2022 G. C.

A multi-stage probability sampling technique was employed. There are five sub-cities in Dessie town, and one sub-city was selected from the entire, employing a simple random sampling technique, then within this chosen sub-city there are three kebeles, and therefore the numbers of participants were selected from each kebele using the proportional allocation of the sample size. Then, a systematic random sampling technique was employed to select study units. the primary study unit was selected randomly between 1st and Kth and study subjects in every Kth household. When more than one study subject was found in one household, a lottery method was used to select a participant. If the chosen house is closed during data collection, the subsequent household participant was interviewed. i.e., K_j_ = N_j_/n_j_ where; K_j_ = sampling interval, N_j_ = total number of households, n_j_ = total number of sample size. There are three kebeles within the Menafesha sub-city and having a total of 3,832 households, K or interval was decided by N/n (*K* = 3832/821 = 4). They interviewed one individual from every four households consecutively (Figure 1).

### Data collection method and technique

Data were collected by face-to-face interviews using a structured questionnaire. PTSD was measured by using a 20-item post-traumatic checklist (PCL-5) with scores ranging from 0 to 80 with a five-point Likert scale (0 = Not at all, 1 = A little bit, 2 = moderately, 3 = Quite a bit, 4 = extremely). A score of ≥ 33 was considered a symptom of PTSD (28). The PCL-5’s validity and reliability have been examined and demonstrated in several nations, including Iraq (Cronbach’s alpha = 0.85) (29), and Zimbabwe (Cronbach’s alpha = 0.92) (30). The internal consistency (Cronbach alpha) of (PCL-5s) in this study was 0.87.

The Patient Health Questionnaire (PHQ-9) was used to assess depression, with a score of 10 or higher indicating depression (31). In several nations, the PHQ-9 validity and reliability have been evaluated and verified for usage in community studies (16). The perceived stress (PSS) scale, which has a 0–40 range, was used to quantify felt life threats. According to the PSS, respondents with scores between 0 and 13 had low perceived stress, those between 14 and 26 had moderate perceived stress, and those between 27 and 40 had high felt perceived stress (8). The internal consistency (Cronbach alpha) of (PHQ-9) and perceived stress scale (PSS) were 0.82, and 0.84, respectively.

The Social Support Scale (The Oslo three-items) (OSSS-3) was used to collect data regarding the strength of social support. It was categorized into three broad categories of social support; 3–8 poor social support, 9–11 moderate social support, and 12–14 strong social support (32). The internal consistency (Cronbach alpha) of Oslo-3 social support was 0.79. Anxiety was measured by a sub scale adapted from the Depression, Anxiety, and Stress Scale (DASS -21) with a score of 8 or more suggesting anxiety symptom (bib1). The WHO student drug-use questionnaire was used to measure substances (34). Socio-demographic factors, substance use history, clinical factors, and trauma-related factors were used on “yes/no” response questionnaires and were operationalized according to different works of literature.

### Data collection procedures

The questionnaire was initially written in English, then translated into Amharic, and finally back into English to ensure uniformity. Additionally, we use standard tools to determine the outcome variable by giving 2 days of training for data collectors and supervisors. In the Hotie sub-city, 5% (*n* = 41) of the participants took part in the pre-test, which aimed to identify any potential issues with the data collection methods and suggest changes to the questionnaire. The principal investigator and supervisor regularly oversaw and assisted the data collectors. Every day during the period of data collection, supervisors and primary investigators verified the data for consistency and completeness.

### Data processing and analysis

Data were checked and cleaned before being entered into the computer system using Epi-data version 3.1, and they were then exported to SPSS version 26 statistical software for additional analysis. The researchers employed frequency, proportion, and other descriptive statistics. To account for potential confounding effects, independent variables with a bivariable model *p*-value of less than 0.25 were added to the multivariable regression model. Hosmer and Lemeshow examined the fitness model to ensure its accuracy. The strength of the association was demonstrated by an odds ratio with a 95% CI for all factors in the multivariable model that had a *p*-value of less than 0.05. The final result was to report the findings in text, a table, or a graph. Tolerance and variance inflation factors were checked to test multicollinearity or to see the unique effect of predictors on outcome variables.

## Results

### Socio-demographic factors of the respondents

A total of 785 participants were involved in this study and the response rate was 95.6%. The mean age (SD) of the participants was 36.01 (± 11.29 years), and the majority of 447 (56.9%) were males. The majority of the participants, 521 (66.4%), 519 (66.1%), and 203 (25.9%) were married, Muslim, and had a University degree and above, respectively. The majority of the community were 249 (31.7%) merchants by occupation. According to the participant’s responses, 460 (58.6%) of respondents earned monthly income ≥ 2166 (ETB) (Table 1).

**TABLE 1:** Distribution of socio-demographic factors of Dessie town residents, Ethiopia, 2022 (*N* = 785).

Variables	Category	Frequency	Percentage (%)
Sex	Male	447	56.9
	Women	338	43.1
Age	18–24	83	10.6
	25–34	297	37.8
	35–44	243	31.0
	> 44	162	20.6
Marital status	Married	521	66.4
	Single	189	24.1
	Widowed	39	4.9
	Divorced	36	4.6
Religion	Orthodox	222	28.3
	Muslim	519	66.1
	Protestant	44	5.6
Educational status	Unable to read and write	114	14.5
	Primary school	109	13.9
	Secondary school (9–12 grade)	171	21.8
	College diploma	188	23.9
	University degree and above	203	25.9
Occupational status	Government employee	224	28.5
	Housewife	111	14.1
	Merchant	249	31.7
	Student	119	15.2
	Others*	82	10.4
Income (ETB)	< 2166	325	41.4
	≥ 2166	460	58.6

Others*:-Retired, NGOs, and Farmer; ETB, Ethiopian birr.

### Clinical related factors of the respondents

According to the current study, 40 (5.1%) of respondents had a history of mental illness. Among participants, 39 (5.0%) respondents had a family history of mental illness and 88 (11.2%) participants reported a history of chronic medical illness. Of the participants, 192 (24.5%), and 262 (33.4%) had depressive symptoms and anxiety symptoms, respectively (Table 2).

**TABLE 2:** Description of Clinical related factors of respondents among Dessie town residents, Ethiopia, 2022 (*N* = 785).

Variables	Category	Frequency	Percentage (%)
History of mental illness	Yes	40	5.1
	No	745	94.9
Family history of mental illness	Yes	39	5.0
	No	746	95.0
History of chronic medical illness	Yes	88	11.2
	No	697	88.8
Depressive symptoms	Yes	192	24.5
	No	593	75.5
Anxiety	Yes	262	33.4
	No	523	66.6

### Substance-related factors of the respondents

**FIGURE 2:**
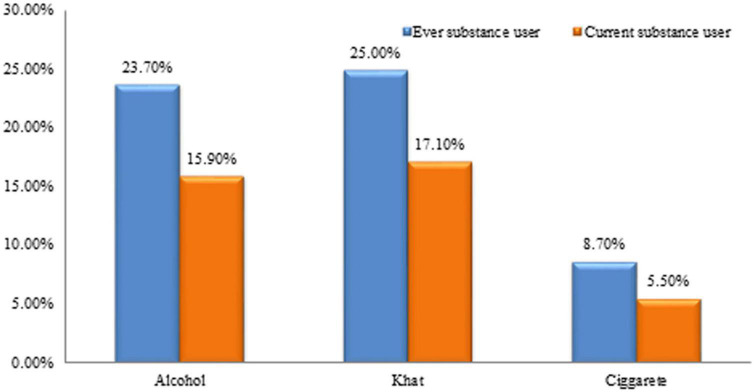
Ever and current substance use among Dessie town residents, Ethiopia, 2022 (*N* = 785).

Regarding the use of the substance, nearly one-fourth 186 (23.7%) had consumed alcohol, 196 (25%) had used khat, and 8.7% had smoked cigarettes in their lifetime. Whereas, 125 (15.9%) consumed alcohol, 134 (17.1%) use khat, and 43 (5.5%) smoke cigarette currently (Figure 2).

### Trauma-related and psychosocial factors

Regarding individual trauma type, the type of trauma experienced by the community 72 (9.2%), 33 (4.2%), 66 (8.4%), and 116 (14.8%) were serious physical injury during the conflict, witness the death of a family member, witness a serious physical injury of a family member or friend, and property destroyed during the conflict, respectively. From this study, 120 (15.3%) had experienced childhood abuse, 357 (45.5%), had moderate perceived life threats and 420 (53.5%) had received threat intermittent social support (Table 3).

**TABLE 3:** Description of Trauma-related and psychosocial factors among Dessie town residents, Ethiopia, 2022 (*N* = 785).

Variables	Category	Frequency	Percentage (%)
Serious physical injury during the conflict	Yes	72	9.2
	No	713	90.8
Witness the death of a family member	Yes	33	4.2
	No	752	95.8
Witness a Serious physical injury of a family member or friend	Yes	66	8.4
	No	719	91.6
Property be destroyed during the conflict	Yes	116	14.8
	No	669	85.2
Childhood abuse history	Yes	120	15.3
	No	665	84.7
Perceived life threat	Low perceived life threat	179	22.8
	Moderate perceived life threat	357	45.5
	High perceived life threat	249	31.7
Social support	Poor social support	176	22.4
	Intermittent social support	420	53.5
	Strong social support	189	24.1

### Prevalence of posttraumatic disorder and associated factors among Dessie town residents

In this study, the overall prevalence of PTSD among people who experienced traumatic events in Dessie town was 19.4% [(95% CI, 16.7, 22.0)].

### Factors associated with posttraumatic stress disorder factors among Dessie town residents

In the bivariate analysis, age, sex, history of mental illness, family history of mental illness, history of chronic medical illness, childhood abuse history, ever alcohol use, current alcohol use, serious physical injury during the conflict, witness the death of a family member, witness a serious physical injury of a family member or friend, the property be destroyed during the conflict, depressive symptoms, perceived life threat, and low social support showed a *P*–value of < 0.25 and became a candidate for multivariable analysis. Variables such as, being female, history of mental illness, depressive symptoms, witnessing a serious physical injury of a family member or friend, and high perceived life threat were found to be significantly associated with PTSD at a *p*-value less than 0.05. in multivariable analysis.

Females were 1.63 times more likely to develop PTSD than males (AOR = 1.63, 95% CI 1.10–2.44). Those participants who had a previous history of mental illness were 3.14 times more likely to develop PTSD as compared with respondents who did not have a history of mental illness (AOR = 3.14, 95% CI 1.14–7.06). Likewise, the odds of developing PTSD among those with depressive symptoms were 3.12 times higher as compared to their counterparts (AOR = 3.12, 95% CI 1.92–5.07). Those who had witnessed a serious physical injury of a family member or friend were 2.82 times more likely to develop PTSD than those who had not witnessed a serious physical injury of a family member or friend (AOR = 2.82, 95% CI 1.18–6.70). Finally, the odds of developing PTSD among participants who had experienced high perceived life threats were five times higher than those who had low perceived life threats (AOR = 5.73, 95% CI 3.05–10.78) (Table 4).

**TABLE 4:** Bivariate and multivariable logistic regression analysis results of posttraumatic stress disorder (PTSD) among Dessie town residents, Ethiopia, 2022 (*N* = 785).

Variables	Category	PTSD		COR (95% C.I)	AOR (95% C.I)	*P*-values
					
		Yes (n)	No (n)			
Age	18–24	16 (19.3%)	67 (80.7%)	1.31 (0.65, 2.62)	1.07 (0.48, 2.35)	0.87
	25–34	60 (20.2%)	237 (79.8%)	1.38 (0.83, 2.31)	0.95 (0.53, 1.68)	0.86
	35–44	51 (21.0%)	192 (79.0%)	1.45 (0.86, 2.46)	1.16 (0.65, 2.07)	0.61
	> 44	25 (15.4%)	137 (84.6%)	1	1	
Sex	Male	74 (16.6%)	373 (83.4%)	1	1	
	Women	78 (23.1%)	260 (76.9%)	1.51 (1.06, 2.16)	1.63 (1.10, 2.44)	0.015*
History of mental illness	Yes	25 (62.5%)	15 (37.5%)	8.11 (4.16, 15.82)	3.14 (1.40, 7.06)	0.006*
	No	127 (17.0%)	618 (83.0%)	1	1	
Family history of mental illness	Yes	24 (61.5%)	15 (38.5%)	7.72 (3.94, 15.14)	1.27 (0.12, 13.35)	0.84
	No	128 (17.2%)	618 (82.8%)	1	1	
History of chronic medical illness	Yes	33 (37.5%)	55 (62.5%)	2.91 (1.81, 4.68)	1.25 (0.59, 2.63)	0.55
	No	119 (17.1%)	578 (82.9%)	1	1	
Childhood abuse history	Yes	41 (34.2%)	79 (65.8%)	2.59 (1.68, 3.97)	0.97 (0.424, 2.23)	0.95
	No	111 (16.7%)	554 (83.3%)	1	1	
Ever alcohol use	Yes	43 (23.1%)	143 (76.9%)	1.35 (0.91, 2.02)	1.32 (0.84, 2.06)	0.23
	No	109 (18.2%)	490 (81.8%)	1	1	
Current alcohol use	Yes	32 (25.6%)	93 (74.4%)	1.55 (0.99, 2.42)	1.18 (0.50, 2.78)	0.702
	No	120 (18.2%)	540 (81.8%)	1	1	
Serious physical injury during the conflict	Yes	31 (43.1%)	41 (56.9%)	3.69 (2.23, 6.14)	1.32 (0.63, 2.75)	0.45
	No	121 (17.0%)	592 (83.0%)	1	1	
Witness the death of a family member	Yes	21 (63.6%)	12 (36.4%)	8.29 (3.98, 17.28)	1.12 (0.25, 4.95)	0.88
	No	131 (17.4%)	621 (82.6%)	1	1	
Witness a serious physical injury of a family member or friend	Yes	35 (53.0%)	31 (47.0%)	5.81 (3.44, 9.79)	2.82 (1.18, 6.70)	0.019*
	No	117 (16.3%)	602 (83.7%)	1	1	
Property be destroyed during the conflict	Yes	45 (38.8%)	71 (61.2%)	3.33 (2.17, 5.10)	1.93 (0.97, 3.83)	0.06
	No	107 (16.0%)	562 (84.0%)	1	1	
Depressive symptoms	Yes	55 (28.6%)	137 (71.4%)	2.05 (1.40, 3.01)	3.12 (1.92, 5.07)	< 0.001*
	No	97 (16.4%)	496 (83.6%)	1	1	
Perceived life threat	Low perceived life threat	15 (8.4%)	164 (91.6%)	1	1	
	Moderate perceived life	49 (13.7%)	308 (86.3%)	1.74 (0.95, 3.19)	1.03 (0.513, 2.06)	0.936
	High perceived life threat	88 (35.3%)	161 (64.7%)	5.97 (3.32, 10.77)	5.73 (3.05, 10.78)	< 0.001*
Social support	Poor social support	42 (23.9%)	134 (76.1%)	1.43 (0.86, 2.37)	1.13 (0.63, 2.02)	0.68
	Intermittent social support	76 (18.1%)	344 (81.9%)	1.01 (0.64, 1.57)	0.99 (0.60, 1.63)	0.97
	Strong social support	34 (18.0%)	155 (82.0%)	1	1	

*Statistically significant at *P*-value < 0.05. COR, Crude odds Ratio; AOR, Adjusted odds Ratio; 1 = reference category, Chi square = 8, Hosmer Lemeshow goodness-of-fit 0.62, degrees of freedom.

## Discussion

War is complex and potentially leads to ever-lasting trauma in the population (27). This population-based study aimed to assess the prevalence of PTSD in northern Etiopia following the civil war. The study showed a higher prevalence of PTSD in the study communities. Additionally, given the high prevalence of PTSD in the study communities, well-coordinated public mental health initiatives should be implemented. Since mental problems can impair people’s ability to operate normally and be productive members of society, reducing the burden of mental disorders, especially (PTSD), is essential (35).

The result of the current study indicated that the magnitude of PTSD among people who experienced traumatic events in Dessie town was 19.4% [(95% CI, 16.7, 22.0)]. The finding was congruent with the study carried out among traumatic Patients in West Ethiopia 17.1% (21), Serbia 18.8% (35), Diyarbakir, Turkey 21.4% (36), and Morocco 19.3% (18). On the other hand, this study’s finding was lower when compared with studies done in North West, Ethiopia 59.8% (16), internally displaced victims in Nigeria 63% (17), a community-based, cross-sectional study landslide survivors, Addis Ababa, Ethiopia 37.3% (22), South Sudan 28% (19), internally displaced people in South Ethiopia 58.4% (20), Palestine 68.9% (37), and Medellin Colombia 88% (26). The disparity could be explained by the population makeup of the participants. For instance, the Palestinian study was restricted to young people who were living in refugee camps during the Al-Aqsa intifada and who had significant injuries as a result of ongoing hostilities. Since the trauma was severe and persisted for a long time, this made the young people more vulnerable. Hence, numerous research has shown that the prevalence of PTSD increases along with the level of exposure to traumatic events, such as the quantity or intensity of the experienced events (16). Other potential causes of this discrepancy include the use of various measurement tools and cut-off points for PTSD, exposure to numerous trauma, study design, and the type and degree of the magnitude of the accidents exposed in the study.

This result was higher than those found in studies in Uganda 11.8% (23), Southern Brazil 9.1% (24), Sri Lanka 2.3% (25), and Sindh 9% (38). The discrepancy in the instruments could be the cause of this variation; in which in Uganda the mini-international neuropsychiatric interview (MINI) was used (23), Composite International Diagnostic Interview (CIDI) in Sri Lanka (25), structured interview using DSM-IV-TR in Sindh (38), whereas in this study the PCL-C was used, and extended standards criteria A which was modified with a better internal consistent in order to measure PTSD (28). Another reason might be the duration of exposure to traumatic events; the study was conducted in Uganda 7 years after the conflict, Sri Lanka was after 20 years of forced displacement, but the current study was conducted after 6 months of exposure to trauma. As a result, the lengthened exposure to the traumatic event was more likely to result in a reduction in magnitude due to recall bias. Furthermore the different types of trauma exposure, the sample methods, and sociocultural aspects. could be a reason for their variation.

Regarding the associated factors, females were 1.63 times more likely to develop PTSD than males. This finding was in agreement with different studies in South Africa (39), and two different studies in Ethiopia (16, 20). This might be because women are more likely than men to experience a lower threshold from exposure to psychotrauma, which increases their chance of developing PTSD (26). It may also be a result of the psychological effects of rape or sexual abuse, violent partner loss, higher rates of poverty, children, and being a single parent or widow than men (35). Another factor might be that females react to stress more emotionally and ruminatively than males do. This could make getting PTSD more likely (25).

In the current study, we found that participants who had a previous history of mental illness were 3.14 times more likely to develop PTSD as compared to those who did not have a history of mental illness. Similar to a finding of different studies from Kenya (40), and South Korea (41). In comparison to participants without a history of mental illness, individuals with a history may have higher neurochemical imbalance and neuronal damage. Therefore, stressful situations that persons who have neuron-chemical imbalance due to a history of psychiatric illness encounter speed up the onset of PTSD (35).

The odds of developing PTSD among those with depressive symptoms were 3.12 times higher as compared to their counterparts. This is similar to findings done in Kenya (40), Maikadra, Ethiopia 59.8% (16), and South Ethiopia (20). This may be due to the fact that participants with depressive symptoms are more likely than responders without depressive symptoms to have experienced traumatic events, which in turn raises the risk of developing PTSD (26). Another factor contributing to the higher likelihood of developing PTSD is having previously experienced depressive symptoms and other psychological problems (25).

This finding also revealed that participants who had witnessed a serious physical injury of a family member or friend were nearly three times more likely to develop PTSD than those who had not witnessed a serious physical injury of a family member or friend. This was supported by the study conducted in Bangladesh (39), and the Wenchuan earthquake in China (42). It is now widely recognized that witnessing traumatic events directly can lead to the development of post-traumatic stress disorder (PTSD). This may be the case because witnessing traumatic events can have effects that are similar to those of other trauma victims, such as re-experiencing the event, intrusive negative thoughts including ideas of retaliation, and it may have a big influence on emotional health (17).

Finally, the odds of developing PTSD among participants who had experienced high perceived life threats were five times higher than those who had low perceived life threats. A current study finding was congruent with a finding from North West, Ethiopia 59.8% (16), Koshe landslide in Addis Ababa, Ethiopia 37.3% (22), and South Korea (41). The onset and persistence of PTSD will be accelerated by negative views about the detrimental implications of the ongoing threat (43).

## Limitations of the study

The use of a high response rate and the inclusion of significant variables that were left out of earlier research. Also, for measuring PTSD, we employed an updated standardized tool even it is not validated in Ethiopia. Additionally, to evaluate independent variables including depressive symptoms and perceived stress, validated and standardized instruments were used. Whereas; we were unable to conclude the causes of the connections we discovered because of the study’s cross-sectional nature. The research could not include the afflicted demographic as children in traumatic events, this is recommended for future researchers to perform their studies among this affected population. In addition, recall bias and social desirability may potentially be further limitations. Because the data was collected through a face-to-face interview, which could influence participants’ responses to socially acceptable questions about substances, people with PTSD symptoms may be more motivated to remember earlier exposure than those without the symptoms.

## Conclusion

The current study showed a high prevalence of PTSD among people who experienced traumatic events in Dessie town. Being female, a previous history of mental illness, depressive symptoms, witnessing a serious physical injury of a family member or friend, and high perceived life threats were found to be significant predictors of PTSD. Therefore, mental health programs by local officials, psychologists, and non-governmental groups should be expanded for screening and providing treatment for all people suffering from PTSD to minimize the prevalence of this condition. In addition, mental health awareness campaigns for trauma victims to seek mental health treatment and intense and persistent psychosocial interventions should be provided.

## Data availability statement

The raw data supporting the conclusions of this article will be made available by the authors, without undue reservation.

## Ethics statement

The studies involving human participants were reviewed and approved by Ethical Committee of Wollo University College of Medicine and Health Science with an ethical review board with an ethical review number (RCSPG-205/14). All study participants were told that participation was completely voluntary, that written informed consent was obtained, and that they could withdraw from the study at any time if they were not comfortable. A participant’s confidentiality and privacy were ensured by not including a personal identifier. All methods were performed in accordance with the relevant guidelines and regulations. The patients/participants provided their written informed consent to participate in this study.

## Author contributions

TA was the principal investigator of the study and involved from inception to design acquisition of data analysis, interpretation, and drafting and preparing of the manuscript. YZ, MS, AA, and NB were involved in the reviewing of the proposal and critical review of the draft manuscript. All authors read and approved the final manuscript.

## Abbreviations

AOR: Adjusted Odds Ratio
CI: Confidence Interval
COR: Crude Odds Ratio
DSM: Diagnostic Statistical Manual
ICD: International Classification of Disease
NGOs: Non-governmental organizations
OSSS-3: Social Support Scale
PCL: Post-traumatic Stress Disorder Checklist
PHQ-9: Patient Health Questionnaire
PSS: Perceived Stress Scale
PTSD: Post-traumatic Stress Disorder
SD: Standard deviation
SPSS: Statistical Package for Social Science
WHO: World Health Organization
YLD: Years of healthy life lost due to disability.
